# IKKβ Activation Is Sufficient for RANK-Independent Osteoclast Differentiation and Osteolysis

**DOI:** 10.1002/jbmr.4

**Published:** 2010-02-01

**Authors:** Jesse E Otero, Simon Dai, Muhammad A Alhawagri, Isra Darwech, Yousef Abu-Amer

**Affiliations:** 1 Departments of Orthopedics and Cell Biology & Physiology, Washington University School of MedicineSt. Louis, MO, USA; 2 Orthopedic Research and Cell Biology & Physiology, Washington University School of MedicineSt. Louis, MO, USA

**Keywords:** IKKβ, osteoclast, RANK, NF-κB, osteolysis

## Abstract

Monocytes differentiate into osteoclasts through stimulation of receptor activator of NF-κB (RANK). Many downstream effectors of RANK play a positive role in osteoclastogenesis, but their relative importance in osteoclast differentiation is unclear. We report the discovery that activation of a single pathway downstream of RANK is sufficient for osteoclast differentiation. In this regard, introduction of constitutively activated IKKβ (IKKβ^SSEE^) but not wild-type IKKβ into monocytes stimulates differentiation of bona fide osteoclasts in the absence of RANK ligand (RANKL). This phenomenon is independent of upstream signals because IKKβ^SSEE^ induced the development of bone-resorbing osteoclasts from RANK and *IKKα* knockout monocytes and in conditions in which NEMO-IKKβ association was inhibited. NF-κB p100 and p105, but not RelB, were critical mediators of this effect. Inflammatory autocrine signaling by tumor necrosis factor α (TNF-α) and interleukin 1 (IL-1) were dispensable for the spontaneous osteoclastogenesis driven by IKKβ^SSEE^. More important, adenoviral gene transfer of *IKKβ*^*SSEE*^ induced osteoclasts and osteolysis in calvariae and knees of mice. Our data establish the sufficiency of IKKβ activation for osteolysis and suggest that IKKβ hyperactivation may play a role in conditions of pathologic bone destruction refractory to RANK/RANKL proximal therapeutic interventions. © 2010 American Society for Bone and Mineral Research.

## Introduction

Bone balance depends on the concerted activity of osteoblasts, bone-forming cells, and osteoclasts, bone-resorbing cells. In pathologic conditions such as rheumatoid arthritis and osteoporosis, bone balance favors increased osteoclast activation,(1) resulting in bone pain and increased fracture risk. Therefore, therapies that target the osteoclast are useful in these conditions.(2) On the other hand, gene mutations that disrupt osteoclast differentiation lead to development of osteopetrotic bones that compromise bone homeostasis.(3) Undoubtedly, increasing understanding of the factors that regulate the osteoclast in health and disease will offer important insight into new therapies for bone loss associated with pathologic conditions and for osteopetrosis.

The osteoclast differentiates from monocyte precursors through the action of ligand for the receptor activator for NF-κB (RANKL) and macrophage colony-stimulating factor (M-CSF).(1) On stimulation of their cognate receptors, RANK and c-Fms, a series of signaling events induces activation of transcription factors such as NF-κB, AP-1, and NFATc1, which results in fusion of precursors and expression of genes required for osteoclast function, including *β*_*3*_*-integrin*, *cathepsin K*, *tartrate-resistant acid phosphatase* (*TRACP*), and *matrix metalloproteinase 9* (*Mmp9*).(4) With expression of all necessary genes for osteoclast differentiation, the ability of the osteoclast to resorb bone requires tight regulation of the actin cytoskeleton. Genetic murine models have revealed a number of proteins whose activity is required for cytoskeletal regulation and bone resorption.(5–9) These molecules contribute to formation of the actin ring, the signature of a polarized, bone-resorbing osteoclast.(10)

The complement of proteins that are important for osteoclast differentiation makes up a growing list, but their relative importance remains unclear. A well-studied family of transcription factors that is required for osteoclastogenesis is NF-κB. NF-κB p100 and p105 are both required together for osteoclast differentiation.(11) Additionally, p65/RelA(12) and RelB(13) have been shown to play complementary roles in osteoclast survival and differentiation, respectively. Therefore, factors that activate NF-κB are logical targets for the treatment of osteoclast-mediated disease.

The IκB kinase (IKK) complex activates NF-κB downstream of RANK. Upstream signals lead to association of two catalytically active kinases, IKKα and IKKβ, with the noncatalytic member IKKγ/NEMO. This association is required for activation of IKK through phosphorylation of two IKK activation-loop serines. IKK then phosphorylates IκB, targeting it for proteasomal degradation and allowing NF-κB to enter the nucleus and regulate gene transcription.(14–16) Pharmacologic inhibition of the IKK association with NEMO abrogates osteoclastogenesis and inflammatory osteolysis.(17,18) Furthermore, mice devoid of IKKα(19) or IKKβ(20,21) demonstrate an impaired ability for osteoclast development in vitro. Moreover, mice lacking IKKβ displayed osteopetrosis and resistance to inflammatory bone erosion, whereas mice lacking active IKKα showed no skeletal phenotype.(20) This finding implicates IKKβ as an important target for therapy in osteoclast-mediated disease.

We now report that IKKβ is not only necessary for RANKL-mediated osteoclastogenesis, but its activation also is sufficient for osteoclast formation. Using retroviral delivery of constitutively active IKKβ (IKKβ^SSEE^), we reveal a signal for differentiation of functional osteoclasts that occurs downstream of, but independent from, RANK. IKKβ^SSEE^, but not wild-type IKKβ or IKKα^SSEE^, induces osteoclast differentiation from monocytes. This phenomenon depends on NF-κB but does not require NEMO, IKKα, or RelB. Finally, adenoviral gene transfer of *IKKβ*^*SSEE*^ in knees and calvariae of mice is sufficient for development of massive osteolysis. Our findings demonstrate for the first time that a single activated kinase is sufficient for RANK-independent osteoclast differentiation and that active IKKβ induces osteolytic disease. These data highlight the centrality of IKKβ in osteoclast differentiation and implicate hyperactivation of IKKβ in pathologic bone destruction.

## Materials and Methods

### Animals and cells

All mice were housed in a controlled barrier facility at Washington University (St Louis, MO, USA). *TRACP-Cre* mice(22) were from Dr Roodman (University of Pittsburgh, PA, USA). *Floxed IKKβ*(23) mice were from Dr Pasparakis (University of Cologne, Germany). *TRACP-Cre floxed/floxed IKKβ* mice were generated by crossing *TRACP-Cre* transgenic mice with *floxed IKKβ* mice. *IKKα* heterozygous mice(24) were obtained from Dr Akira (Osaka University, Japan). *RelB* knockout(25) and control bone marrow was from Dr Novack (Washington University, St Louis, MO, USA). *RANK* knockout(26) and control spleens, as well as *NF-κB* double-knockout(27) and control spleens were provided by Dr Xing (University of Rochester Medical Center, Rochester, NY, USA) For in vivo experiments, wild-type C57BL/6 mice at 5 to 6 weeks of age were used.

### Plasmids

pMxs retroviral expression plasmid was from Dr T Kitamura (University of Tokyo, Japan). Mouse cDNA for *IKKα* was kindly provided by Dr Kenneth Marcu (Stony Brook, NY, USA). *IKKβ* and *RelB* cDNA were purchased from ATCC (Manassas, VA). *RelA* cDNA was provided by Dr C Sasaki (NIA, Baltimore, MD, USA). All expression constructs were subcloned into pMxs using standard techniques. The following mutations were generated using the QuickChange II Site Directed Mutagenesis Kit (Stratagene, La Jolla, CA, USA) with primer pairs in parentheses: *IKKβ*^*SSEE*^ (IKKβ_S177_181E_f, GAGCTGGATCAGGGCGAACTGTGCACGGAATTTGTGGGGACTCTGC, and IKKβ_S177_181E_r, GCAGAGTCCCCACAAATTCCGTGCACAGTTCGCCCTGATCCAGCTC); *IKKβ*^*WWAA*^ (IKKβ_W739_741A_f, GACTCTAGACGCGAGCGCGTTACAGATGGAGGATG, and IKKβ_W739_741A_r, CATCCTCCATCTGTAACGCGCTCGCGTCTAGAGTC); *IKKβ*^*KM*^ (IKKβ_K44M_f, GTGAACAGATCGCCATCATGCAATGCCGACAGGAGC, and IKKβ_K44M_r, GCTCCTGTCGGCATTGCATGATGGCGATCTGTTCAC); and *IKKα*^*SSEE*^ (IKKα_S176_180E_f, GATGTTGATCAAGGAGAGCTCTGTACAGAATTTGTGGGAACATTGC, and IKKα-S176_180E_r, GCAATGTTCCCACAAATTCTGTACAGAGCTCTCCTTGATCAACATC). Note that the constitutive activating effect of the mutation of *IKKβ* was established previously.(28,29)

### Generation of monocytes/macrophages

Marrow was flushed from long bones into α minimum essential medium (α-MEM). Spleens and day 18.5 fetal livers were crushed into cell suspensions in α-MEM and were centrifuged at 453 rcf. Cell pellets were resuspended in whole medium [α-MEM with 1× penicillin/streptomycin, 10% heat-inactivated fetal bovine serum (FBS)]. Monocytes/macrophages were produced by growing cell suspensions in the presence of 10 ng/mL of M-CSF. Monocytes/macrophages were allowed to proliferate for 3 days at 37°C in 5% CO_2_, at which point they were infected with retrovirus (50% virus supernatant, 50% α-MEM containing 10% FBS, 10 ng/mL of M-CSF, 100 U penicillin/100 µg strep per Liter, and 4 µg/mL hexadimethrine bromide). Twenty-four hours after infection, cells were selected in α-MEM containing 10% FBS, 10 ng/mL of M-CSF, 100 U penicillin/100 µg strep per Liter, and 2 µg/mL puromycin for 72 hours, at which point selection medium was removed, and cells were washed and grown for 24 additional hours without puromycin. At this point, cells were lifted, counted, and plated for downstream experiments.

### Generation of retrovirus

The use of Plat-E retrovirus packaging cells stably expressing retroviral structural proteins gag-pol and env for transient production of high-titer retrovirus was described previously.(30) Briefly, 8 µg of pMx vectors expressing our gene of interest was transfected into 5 million Plat-E cells (grown in DMEM supplemented with 10% FBS, 10 ng/mL of M-CSF, and 100 U penicillin/100 µg strep per Liter) using Fugene 6 (Roche, Palo Alto, CA, USA) according to manufacturer's instructions. Twenty-four hours after transfection, the medium was exchanged to remove the transfection reagent. Then 24 and 48 hours after medium exchange, supernatant was collected and pooled for infection of monocytes (see above).

### In vitro osteoclastogenesis

For osteoclastogenesis assays, 3 × 10^4^ monocytes were plated in 200 µL of α-MEM with 10% FBS. IKKβ^SSEE^-expressing cells were cultured in 10 ng/mL of M-CSF, whereas GFP*-* and IKKβ^WT^-expressing cells were cultured in 10 ng/mL of M-CSF plus 100 ng/mL of RANKL for 4 days. At this point, cells were fixed and TRACP stained using the Leukocyte Acid Phosphatase Kit (Sigma, St Louis, MO, USA). TRACP^+^ cells with three or more nuclei were scored as osteoclasts.

### Inhibitor studies

For inhibition of osteoclastogenesis, cells were treated with 100 ng/mL of *OPG/Fc* chimera (R&D Systems, Minneapolis, MN, USA), 25 µM TAT-NBD (YGRKKRRQRRR-G-TTLDWSWLQME) or 25 µM of TAT–mutant NBD (YGRKKRRQRRR-G-TTLDASALQME) during the entire course of retroviral transduction and in vitro osteoclast differentiation.

### RNA isolation and cDNA production

RNA was isolated from macrophage or osteoclast cultures using the Total RNA Isolation Mini Kit (Agilent Technologies, Santa Clara, CA, USA) according to manufacturer's instructions. Reverse transcription was described previously.(21)

### Real-time quantitative PCR

The real-time quantitative PCR (qPCR) procedure was described in detail previously.(21)

### Western blotting

The Western blot procedure was described previously.(21) One million cells were used for protein extraction and demonstration of protein expression.

### Coimmunoprecipitation

One million cells expressing GFP, flag IKKβ^WT^, flag IKKβ^WA^, flag IKKβ^SSEE^, or flag IKKβ^SSEE/WA^ were lysed in immunoprecipitation (IP) buffer [10 mM Tris, pH 7.4, 150 mM NaCl, 0.5% NP-40 (IGEPAL), 1 mM EDTA, 1 mM NaF, 1 mM PMSF, 1 mM Na_3_VO_4_, and 1× protease inhibitor cocktail] at 4°C. Protein was measured by BCA Assay (Pierce, Rockford, IL, USA) and normalized. Nonspecific binding was removed by rocking total cell lysate at 4°C with GammaBind G Sepharose beads (GE Lifesciences, Piscataway, NJ) and 100 ng of normal mouse IgG for 2 hours at 4°C. Beads and normal antibody were centrifuged, and supernatant was incubated with GammaBind G Sepharose beads and 1 µg/mL of mouse anti-Flag M2 antibody (Sigma) in a total of 700 µL of IP buffer and 1× protease inhibitor cocktail at 4°C for 16 hours. Immune complexes were centrifuged with beads. Supernatant was removed by vacuum suction, and 2× sample buffer [0.5 M Tris-HCl, pH 6.8, 10% (w/v) SDS, 10% glycerol, 0.05% (w/v) bromphenol blue, 3% β-mercaptoethanol, and distilled water] was added to the beads, which were boiled for 5 minutes to elute the complex components, which were analyzed by Western blot.

### Kinase assay

Plat-E cells expressing indicated flag-tagged IKKβ constructs were lysed in IP buffer. IKKβ was immunoprecipitated with M2 antibody, washed twice with IP buffer and once with kinase assay buffer (Cell Signaling Technologies Danvers, MA, USA), and incubated for 30 minutes at 30°C in 30 µL of kinase assay buffer with 1 µg GST-IκBα, 2.5 mM MgCl_2_, and 16 µM ATP. The reaction was terminated with 30 µL of reducing sample buffer. Samples were analyzed by Western blot.

### Bone-resorption assays

Osteoclasts were cultured on 5 mm^2^ 100-µm-thick dentin slices for 5 days in a 48-well tissue culture plate. To visualize resorption pits and tracks, slices were exposed to 0.5 N NaOH, and cells were removed by mechanical agitation. Slices were washed in PBS and stained with 0.1% toluidine blue (w/v) in PBS. Stained slices were rinsed with PBS and blotted dry, and pits were visualized by light microscopy. Resorption of artificial matrix was described previously.(21)

### Actin ring staining

Cells were fixed in 4% paraformaldehyde in PBS for 5 minutes at room temperature. Fixed macrophages or osteoclasts on dentin slices were washed with PBS and permeabilized in 0.2% Triton X-100 in PBS for 10 minutes at room temperature. Dentin slices were washed with PBS and then incubated in a 1:40 dilution of Alexa Fluor-488 phalloidin (Invitrogen Molecular Probes, Eugene, OR, USA) for 10 minutes in a dark, humidified chamber at room temperature. Slices were washed with PBS and mounted onto microscope slides for visualization of actin rings with fluorescent microscopy.

### Generation and use of adenovirus

Adenovirus expressing *IKKβ*^*SSEE*^ was generated by subcloning from the pMx parental vector into Ad5 CMV K-NpA Shuttle using EcoR1 and Not1 restriction endonucleases (New England Biolabs, Ipswich, MA, USA). Recombination,(31) production, and characterization [plaque-forming units (pfus)/particle] of virus were provided by Viraquest, Inc. (North Liberty, IA, USA). For local in vivo gene transfer in mice, 1 × 10^7^ pfus of virus diluted in 10 µL of sterile PBS were injected intraarticularly into the knee joint capsule. Contralateral knees on the same mouse served as experimental (Ad IKKβ^SSEE^) and control (Ad ntLacZ). Five mice were used for these experiments with comparable results. For calvarial osteolysis, 1 × 10^7^ pfus of virus diluted in 50 µL of sterile PBS were injected supracalvarially. Lipopolysaccharide (LPS) (10 µg) or RANKL (4 µg) diluted in 50 µL total volume of PBS were injected as positive controls. For calvarial experiments, five mice were used for each condition (virus, RANKL, and LPS) with comparable results. In vivo injections were executed with 100-mL insulin syringes with 29G needles. Seven days after injection, knees and calvariae were fixed, decalcified, and analyzed histologically for osteoclasts and osteolysis.

### Histology

Bones were collected from mice and fixed in 10% buffered formalin for 24 hours. Bones then were decalcified for 7 days in buffer consisting of [14% (w/v) EDTA and H_4_NOH, pH 7.2], dehydrated in ethanol (30% to 70%), cleared through xylene, and embedded in paraffin. Sections were stained histochemically for TRACP to visualize osteoclasts or hematoxylin and eosine (H&E) to assess tissue architecture. Immunohistochemistry was performed according to the antibody manufacturer's instructions for immunoperoxidase staining.

### Microscopy

Cells and histologic sections were imaged under white or ultraviolet (UV) light on an inverted microscope (Olympus IX-51). For f-actin visualization, UV light was passed through an FITC filter cube to localize green phalloidin. Digital images were captured using a CCD camera (Olympus DP70, 12-megapixel resolution).

### Statistics

Student's two-tailed *t* test for comparison between means was used for all analyses.

## Results

### Constitutively active IKKβ induces RANKL-independent osteoclast differentiation from monocytes

**Fig. 1:**
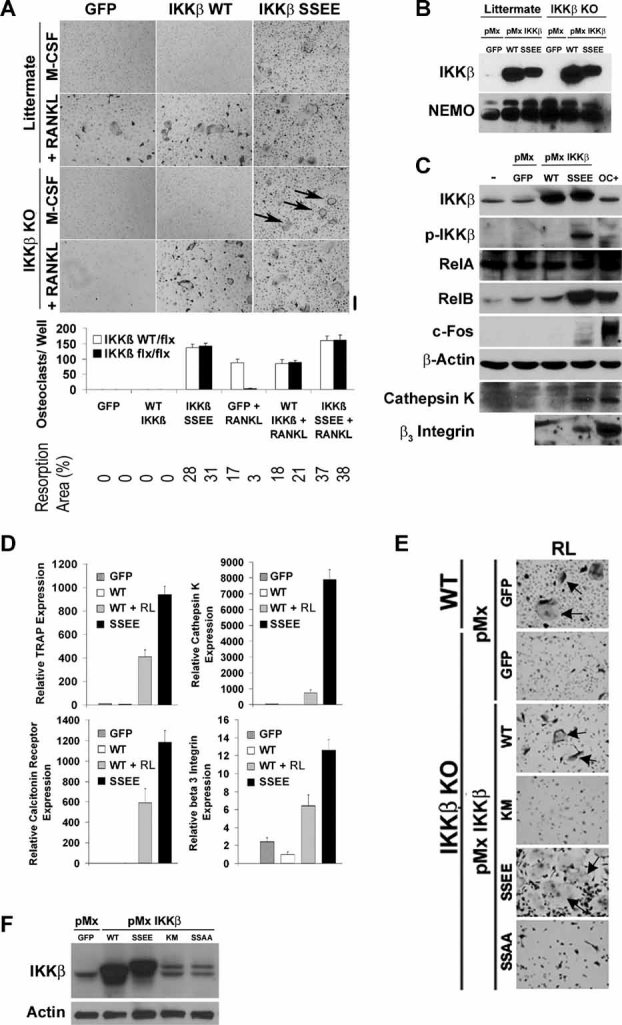
IKKβ^SSEE^ induces bona fide osteoclasts from bone marrow, spleen, and fetal liver progenitors. (*A*) Bone marrow macrophages from *IKKβ* knockout and littermate mice were infected with retroviruses expressing GFP, IKKβ^WT^, or IKKβ^SSEE^ and cultured with M-CSF alone or M-CSF + RANKL for 4 days (as described under “Materials and Methods”) and were TRACP stained to visualize osteoclasts. Arrows denote osteoclasts. Lower panels, quantification; flx = loxP flanked. For bone resorption, fetal liver cells (FLCs) were plated on dentin slices to determine bone resorption. After 5 days, slices were stained with toluidine (Tol.) blue and phalloidin to visualize resorption tracks (*darker areas*) and actin ring formation, respectively. Cells were cultured in M-CSF alone except where indicated. Quantification of percent resorbed area was done using Bioquant. Percent resorption area of dentin slices for each condition is denoted. (*B*) Expression levels of IKKβ^WT^ and IKKβ^SSEE^ in control and *IKKβ* knockout macrophages were measured by Western blot using anti-IKKβ antibody. Expression of endogenous NEMO is shown as control. (*C*) Spleen cells were plated as detailed under “Materials and Methods.” After 3 days, cells were infected with the various pMx viruses as indicated. Cells expressing the viral proteins were selected with puromycin for 2 to 3 days. Western blot for expression of NF-κB molecules and osteoclast markers in total cell lysates of spleen cells infected with the indicated viruses was performed using equal amounts of total proteins. Actin expression indicates equal loading. OC+ = osteoclast positive control total cell lysate. (*B*, *C*) pMx = retroviral expression vector. (*D*) Relative expression of mRNA for osteoclast markers by real-time qPCR. mRNA was extracted from cells infected with the indicated viruses. Relative expression of the indicated osteoclast marker genes was determined using specific primers outlined under “Materials and Methods.” *GAPDH* served as internal standard for cDNA normalization. Values are expressed as relative quantity plus standard error of the mean. (*E*) Control and *IKKβ* knockout monocytes were transduced with viruses expressing GFP or the indicated forms of IKKβ [active (SSEE) and inactive (KM, SSAA) forms]. These cells were treated with M-CSF and RANKL and TRACP stained. (*F*) Western blot to demonstrate expression of the indicated IKKβ constructs. Parallel cells treated as shown in panel *E* were lysed and subjected to Western blots with IKK and actin antibodies.

We and others have demonstrated the necessity for IKKβ in osteoclast differentiation.(20,21) In an effort to identify mutations in *IKKβ* that could prevent or enhance its ability to rescue osteoclast differentiation in cells lacking IKKβ, we made the observation that constitutively activated IKKβ (IKKβ^SSEE^), but not the wild-type (IKKβ^WT^) form, in wild-type or *IKKβ* knockout bone marrow–derived macrophages induced the formation of osteoclasts in the absence of RANKL (Fig. 1*A*). Levels of IKKβ^WT^ and IKKβ^SSEE^ protein were comparable (Figure 1B), whereas IKKβ^SSEE^, but not IKKβ^WT^, was recognized by an antibody specific for IKKβ phosphorylated at activation-loop serines (Fig. 1*C*), suggesting that the kinase domain of IKKβ^SSEE^ exists in an active conformation and that mutation of IKKβ activation-loop serines 177 and 181 to glutamic acid, and not overexpression of IKKβ, is responsible for the formation of osteoclasts in the absence of RANKL.

Further characterization showed that IKKβ^SSEE^ induced expression of RelB and c-fos, which are important for normal osteoclast differentiation.(13,32) IKKβ^SSEE^, but not IKKβ^WT^, also induced the expression of β_3_-integrin and cathepsin K, two markers for mature osteoclasts whose products are required for bone resorption(6,33) (Fig. 1*C*). Real-time qPCR analysis revealed that IKKβ^SSEE^ also induced expression of calcitonin receptor, cathepsin K, TRACP, and β_3_-integrin (Fig. 1*D*). Furthermore, IKKβ^SSEE^-induced osteoclasts form actin rings and resorb artificial (not shown) and authentic bone matrix (Fig. 1*A*). Expression of IKKβ^SSEE^ by RANKL-independent osteoclasts was demonstrated using IKKβ^SSEE^-GFP fusion construct (not shown). These data provide evidence that the TRACP^+^ multinucleated cells induced through expression of constitutively active IKKβ in macrophages are authentic osteoclasts.

Next, we sought to examine whether stimulation of osteoclast differentiation through introduction of IKKβ^SSEE^ was a phenomenon restricted to precursors obtained from adult tissue. To this end, IKKβ^SSEE^- infected, but not GFP- or IKKβ^WT^-infected, fetal liver cells formed authentic osteoclasts with visible actin rings that resorbed dentin (not shown). Actin rings and resorption pits were observed in IKKβ^WT^-infected cells only after RANKL administration (not shown). These observations reveal that IKKβ^SSEE^ is sufficient to induce an authentic program for functional osteoclasts from adult and fetal precursor cells independent of RANKL. To verify the specificity of the osteoclastogenic effect of the phosphomimmetic mutation, we mutated IKKβ activation-loop serines to alanine (IKKβ^SSAA^) and lysine to methionine (IKKβ^KM^), respectively. These mutations resulted in an activation-deficient molecule that failed to rescue RANKL-induced osteoclastogenesis in *IKKβ* knockout monocytes (Fig. 1*E, F*). Therefore, phosphomimmetic mutation of IKKβ activation-loop serines is a specific inducer of the osteoclast program, and inactivating the kinase domain of this molecule hinders its osteoclastogenic activity.

### IKKβ^SSEE^ Rescues *RANK* Knockout Osteoclast Phenotype

**Fig. 2:**
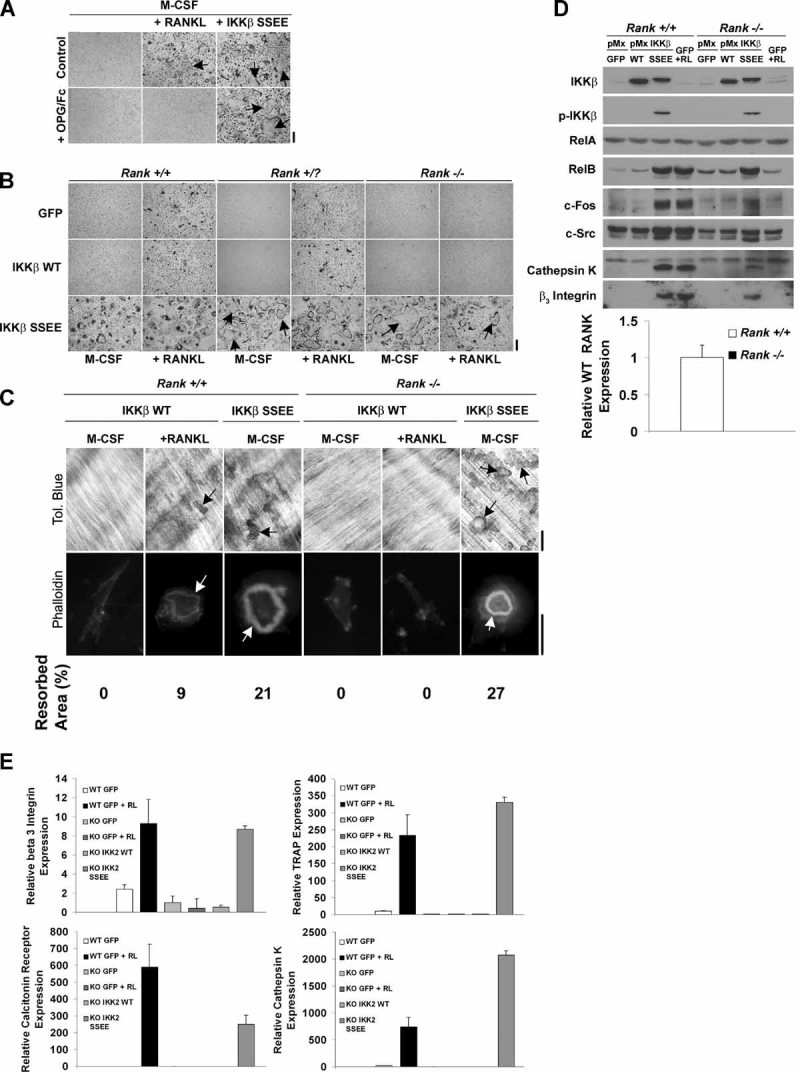
IKKβ^SSEE^-induced osteoclastogenesis does not require RANKL/RANK upstream signals. (*A*) Equal number of macrophages (30,000 cells/well) were cultured in the presence of M-CSF with or without RANKL, each in the absence or presence of OPG/Fc chimera. IKKβ^SSEE^- expressing cells were cultured with M-CSF in the absence or presence of OPG/Fc chimera. Cultures were carried out as described under “Materials and Methods.” Cells were stained with TRACP to visualize osteoclasts. (*B*) Wild-type, *RANK* ^+/?^, or *RANK*^–/–^ spleen-derived macrophages were infected with a retrovirus expressing GFP, IKKβ^WT^, or IKKβ^SSEE^. These cells were cultured in the presence of M-CSF alone or in combination with RANKL for 4 days and stained with TRACP to visualize osteoclasts. (*C*) Wild-type and *RANK^−/−^* spleen-derived macrophages were infected with a retrovirus expressing IKKβ^WT^ or IKKβ^SSEE^. These cells were cultured in the presence of M-CSF alone or in combination with RANKL on dentin and were stained with phalloidin or toluidine (Tol.) blue to visualize actin rings and resorption pits, respectively. Scale bars indicate relative magnification. Resorbed areas were quantified using Bioquant and expressed as percent area. (*D*) An equal number of wild-type (+/ + ) or *RANK* knockout (−/−) spleen cells infected with the indicated viruses were cultured in the presence of M-CSF or M-CSF + RANKL (RL), and an equal number of total cell lysates were analyzed by Western blot for expression of the indicated proteins. The lower panel represents real-time qPCR for *RANK* mRNA in wild-type and *RANK* knockout cells. (*E*) Relative expression of mRNA for osteoclast markers assessed by real-time qPCR. *GAPDH* served as internal standard normalization. Values are expressed as relative quantity plus SEM.

Having established that RANKL is dispensable for IKKβ^SSEE^-mediated osteoclastogenesis, we tested whether intrinsic RANK signaling played a role in this phenomenon by using the RANKL decoy molecule osteoprotegerin (OPG-Fc)(10,34) and *RANK*-null cells. OPG-Fc completely inhibited RANKL-induced osteoclastogenesis in IKKβ^WT^ -infected macrophages but had no effect on IKKβ^SSEE^-induced osteoclast differentiation, indicating that IKKβ^SSEE^ induces osteoclastogenesis without RANKL (Fig. 2*A*). Furthermore, IKKβ^SSEE^, but not IKKβ^WT^ nor GFP, induced the formation of osteoclasts from *RANK* knockout cells (Fig. 2*B*). Importantly, *RANK* knockout macrophages expressing GFP or IKKβ^WT^ failed to form osteoclasts in response to RANKL (Fig. 2*B*). IKKβ^SSEE^- induced osteoclasts also formed actin rings and resorbed dentin (Fig. 2*C*). Consistent with this result, Western blot revealed that IKKβ^SSEE^ introduction into, but not RANKL treatment of, *RANK* knockout cells resulted in expression of *c-fos* and *RelB*, as well as *c-src*, *β*_*3*_*-integrin*, and *cathepsin K* (Fig. 2*D*), indicating that IKKβ^SSEE^-induced *RANK* knockout osteoclasts are indeed bona fide osteoclasts. Real-time qPCR supported this conclusion (Fig. 2*E*). Therefore, IKKβ^SSEE^ functions independent of RANK to induce differentiation of functional osteoclasts.

### IKKβ^SSEE^ acts independently of the classical IKK complex to drive osteoclastogenesis

**Fig. 3:**
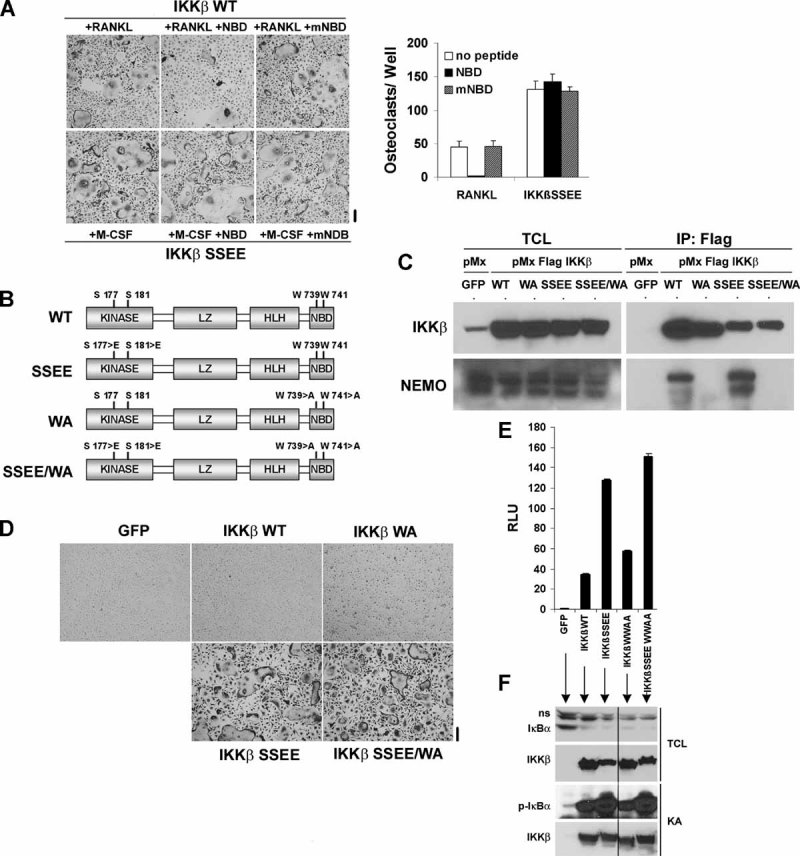
IKKβ^SSEE^ induces osteoclastogenesis independent from NEMO association. (*A*) Bone marrow macrophages were transduced with IKKβ^SSEE^ in the continuous presence of NBD, mutant NBD (mNBD), or vehicle. Cells expressing IKKβ^WT^ were treated with RANKL in the absence of NBD or in the presence of NBD or mNBD. After 4 days, cells were stained with TRACP. Right panel shows quantification of osteoclasts per well from quadruplicate wells and four independent experiments. (*B*) Schematic diagram of IKKβ constructs used in panels *C* and *D*. Kinase = kinase domain; LZ = leucine zipper; HLH = helix-loop-helix; NBD = NEMO-binding domain. Not shown to scale. (*C*) Cells transduced with the various IKKβ forms were lysed. A portion of the lysates was used as a total cell lysate (TCL) to examine expression of IKK and NEMO proteins. Another portion of the lysate was precleared with gamma beads and subjected to immunoprecipitation using anti-Flag antibody. The coimmunoprecipitation of IKK and NEMO depicts the ability of tryptophan 739 and 741 to alanine mutations to prevent binding of IKKβ^WT^ and IKKβ^SSEE^ to NEMO (as shown in lanes annotated *WA* and *SSEE/WA*). (*D*) Cells transduced with the indicated IKKβ forms (shown in panel *B*) were plated in the absence of RANKL for 4 days. Cells then were stained with TRACP to detect osteoclasts. IKKβ^SSEE^ and IKKβ^SSEE/WA^ generated comparable numbers of osteoclasts per well (157 and 169 cells, respectively). (*E*) Luciferase assay for NF-κB induction by constructs shown in panel *B*. Cells were transduced with the indicated IKK forms, and lysates were subjected to luciferase assay, as described under “Materials and Methods.” (*F*) Lysates identical to those shown in panel *E* were subjected to IKK kinase. Expression levels of IκB, phosphorylated IκBα (p-IκBα), and IKKβ are shown.

Activation of IKKβ by upstream signals requires its association, via two carboxyl-terminal tryptophans (W739 and W741), with NEMO.(35,36) Since IKKβ^SSEE^ induces osteoclastogenesis independent of RANK, we tested whether IKKβSSEE also could induce osteoclastogenesis in the absence of NEMO binding. First, we determined that while administration of cell-permeable NBD peptides, which inhibit the association of IKKβ with NEMO, blocks RANKL-induced osteoclast differentiation, NBD did not inhibit osteoclastogenesis in response to transduction of IKKβ^SSEE^ (Fig. 3*A*). Second, while mutations of W739 and W741 to alanine in the presence of the S177 and S181 to glutamic acid (IKKβ^SSEE/WA^) prevent IKKβ^SSEE^ from binding to NEMO (Fig. 3*B, C*), IKKβ^SSEE/WA^ is still capable of inducing RANKL-independent osteoclastogenesis (Fig. 3*D*). This quadruple IKK mutant is expressed properly and retains its kinase activity (Fig. 3*E, F*). These results solidify the conclusion that IKKβ^SSEE^ induces RANKL-independent osteoclastogenesis without binding to NEMO, uncoupling the mechanism of IKKβ^SSEE^-induced osteoclastogenesis from all known upstream stimuli important for osteoclast differentiation. This suggests that in the setting of osteoclast differentiation, IKKβ binding to NEMO is important only for IKKβ activation-loop phosphorylation, after which point the association is not required.

**Fig. 4:**
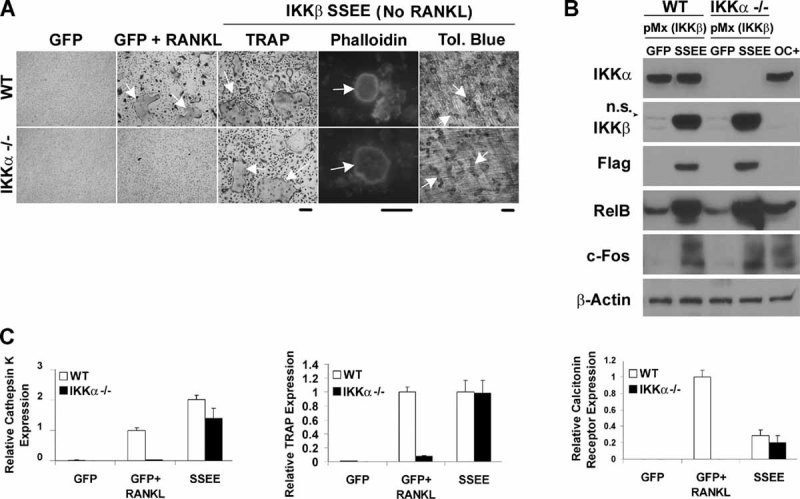
IKKβ^SSEE^ induction of osteoclasts does not require IKKα. (*A*) Wild-type and *IKKα* null FLCs expressing GFP were cultured in M-CSF with or without RANKL or IKKβ^SSEE^ with M-CSF alone. TRACP stain was used for osteoclasts, phalloidin stain for actin rings, and toluidine (Tol.) blue stain for dentin resorption. Wild-type cells infected with IKKβ^SSEE^ generated 133 ± 22 osteoclasts per well compared with 112 ± 12 osteoclasts per well in *IKKβ1* null cells infected with IKKβ^SSEE^. Percent resorbed area was 22% ± 5& and 21& ± 7%, respectively. Scale bars indicate magnification of actin image. (*B*) Western blot for expression of NF-κB pathway markers. OC^+^ = osteoclast-positive control; n.s. = nonspecific band. (*C*) Relative expression of mRNA for markers of osteoclastogenesis by real-time qPCR. Levels normalized to *GAPDH*.

Based on these results, we hypothesized that IKKβ^SSEE^ could induce osteoclastogenesis without the classical IKK complex, which includes IKKα, a kinase that is required for osteoclastogenesis in vitro.(19) We confirmed that *IKKα* null fetal liver–derived macrophages (FLCs) do not differentiate into osteoclasts (Fig. 4*A*) and fail to express mRNA for osteoclast markers in response to RANKL stimulation (Fig. 4*C*). However, transduction of *IKKα* null FLCs with IKKβ^SSEE^ restores osteoclastogenesis in the absence of RANKL, restores actin rings and bone resorption (Fig. 4*A*, phalloidin and tol. blue, respectively), and induces expression of typical signaling proteins (Fig. 4*B*) and expression of mRNA for osteoclast markers (Fig. 4*C*). These data indicate that formation of the classical IKK complex and the IKKα-mediated noncanonical NF-κB signaling pathway are not a requirement for IKKβ^SSEE^ to stimulate RANK-independent osteoclastogenesis.

### Requirement for coordinated NF-κB Activation in IKKβ^SSEE^-Induced Osteoclastogenesis

**Fig. 5:**
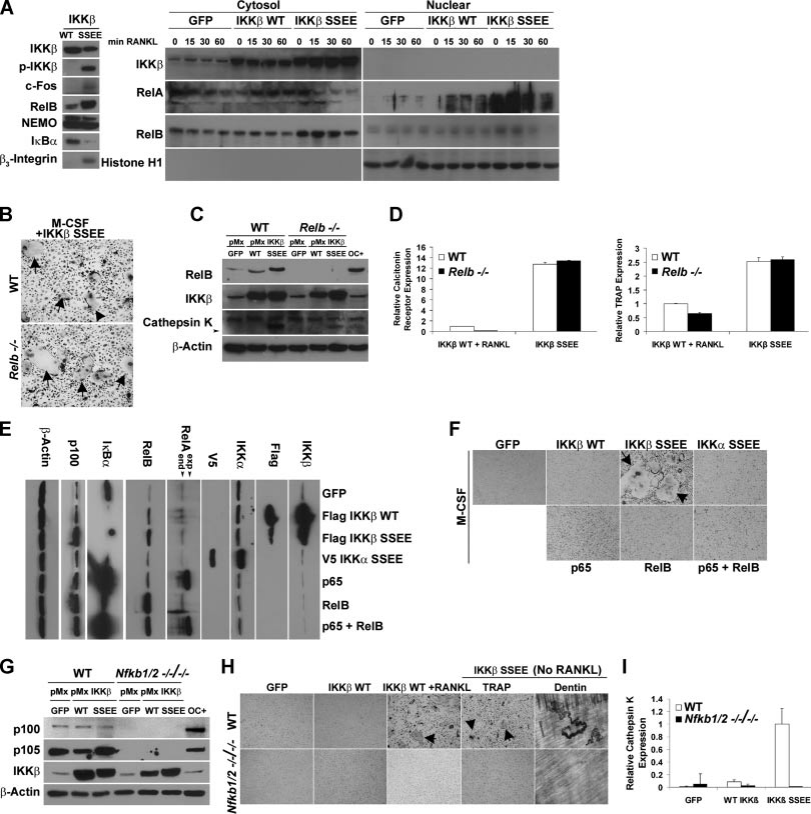
IKKβ^SSEE^ induction of osteoclastogenesis requires coordinated NF-κB signaling. (*A*) Western blot for expression of NF-κB signaling molecules in total cell lysates (*left panel*) and cytosol and nuclear fractions (*right panel*) of spleen macrophages expressing GFP, IKKβ^WT^, or IKKβ^SSEE^ not treated or treated with RANKL for the indicated times. (*B*) TRACP stain of wild-type and *RelB* knockout cells expressing IKKβ^SSEE^ cultured in the presence of M-CSF without RANKL (osteoclasts are marked with *arrowheads*). Number of osteoclasts for wild-type cells was 115 ± 21 cells per well compared with 101 ± 28 cells per well for *RelB* knockout cells. (*C*) Western blot for the indicated proteins in wild-type and *RelB* knockout cells expressing GFP, IKKβ^WT^, or IKKβ^SSEE^ cultured with M-CSF. OC^+^ = osteoclast-positive control. (*D*) Relative mRNA expression of selected osteoclast markers in wild-type and *RelB* knockout cells treated with RANKL or expressing IKKβ^SSEE^ not treated with RANKL. (*E*, *F*) Protein expression and osteoclastogenesis, respectively, were measured following expression of GFP, IKKβ^WT^, IKKβ^SSEE^, IKKα^SSEE^, p65 (RelA), RelB, or p65 + RelB in bone marrow macrophages cultured with M-CSF. (*G*–*I*) Control or *NF-κB* double-knockout spleen cells transduced with GFP, IKKβ^WT^, or IKKβ^SSEE^ were analyzed to determine (*G*) protein expression levels, (*H*) osteoclastogenesis and bone resorption, and (*I*) *cathepsin K* mRNA expression. Cells in panel *I* were cultured in M-CSF without RANKL.

To identify the mechanism underlying IKKβ^SSEE^-induced osteoclastogenesis, we examined the status of essential NF-κB subunits compared with RANKL-treated conditions. We observed elevated levels of RelB in the cytosol of IKKβ^SSEE^-expressing cells at all time points assessed, including nonstimulated, compared with GFP- and IKKβ^WT^-expressing cells. We also observed reduced levels of IκBα that coincided with an increased level of RelA protein in the nucleus in the absence of RANKL stimulation and at all time points tested in IKKβ^SSEE^- compared with GFP- and IKKβ^WT^-expressing cells, indicating that the constitutively activated form of IKKβ induces continuous IκBα processing (Fig. 5*A*). These data suggest that IKKβ^SSEE^ acts through an NF-κB-dependent mechanism to induce osteoclast differentiation. To test this, we challenged *RelB* knockout cells with IKKβ^SSEE^ because we observed induction of RelB protein expression in response to IKKβ^SSEE^ in macrophages and because RelB expression is required for RANKL-induced osteoclast differentiation in vitro and for stimulated but not basal osteoclast formation in vivo.(13) *RelB* knockout bone macrophages were capable of differentiating into TRACP^+^ osteoclasts that express cathepsin K in the absence of RANKL when expressing IKKβ^SSEE^ (Fig. 5*B, C*). Real-time PCR revealed that while induction of expression of mRNA for *calcitonin receptor* and *TRACP* in response to RANKL was impaired in *RelB* null cells, IKKβ^SSEE^ rescued the induction to levels equivalent to that in wild-type cells expressing IKKβ^SSEE^ (Fig. 5*D*). Therefore, IKKβ^SSEE^ does not require RelB to induce osteoclast differentiation.

Given the observation that IKKβ^SSEE^ induces nuclear translocation of RelA and induces increased expression of RelB, we further explored the possibility that these NF-κB subunits mediate the IKKβ^SSEE^ effect. However, overexpression of RelA, RelB, or a combination of RelA and RelB (Fig. 5*F*) did not induce osteoclast differentiation. To verify activity of the RelA and RelB, we observed that RelA induced expression of IκBα and that RelA and RelB alone or in combination induced expression of p100 (Fig. 5*E*). These results indicate that IKKβ^SSEE^ is a specific activator of NF-κB capable of inducing osteoclast differentiation and that ectopic overexpression of RelA and RelB is insufficient to coordinate this effect.

Phosphorylation of T-loop residues is a hallmark of activation for many kinases.(37) Given the specificity of IKKβ activation as a mediator of osteoclast differentiation, we asked whether constitutive activation of other kinases through phosphomimmetic mutations also could induce osteoclast differentiation. IKKα and IKKβ share significant primary and secondary structural homology,(15) so we reasoned that in contrast to other kinases, constitutive activation of IKKα through phosphomimmetic mutation would be most likely to induce an osteoclast program like IKKβ^SSEE^. We found that when expressed at comparable levels (Fig. 5*E*), IKKβ^SSEE^ induces osteoclast differentiation from macrophages, whereas IKKα^SSEE^ had no such effect (Fig. 5F), demonstrating that IKKβ is the specific kinase activator of the osteoclast program.

It has been established that a combination of both NF-κB1/p50 and NF-κB2/p52 subunits is required for osteoclast differentiation.(11) We tested whether IKKβ^SSEE^-induced RANK-independent osteoclastogenesis also requires NF-κB1 and -2 by transducing control and *NF-κB1*^*−/−*^/*NF-κB2*^*−/−*^ (*NF-κB* double-knockout) spleen macrophages with GFP, IKKβ^WT^, and IKKβ^SSEE^ (Fig. 5*G*) and performing TRACP staining for osteoclasts in the absence of RANKL administration. While control cells expressing IKKβ^SSEE^ produced a significant number of osteoclasts capable of resorbing bone coinciding with expression of mRNA for *cathepsin K*, no osteoclasts were observed in *NF-κB* double-knockout cells (Figure 5H,I) despite constitutive IκBα processing (not shown). We conclude that IKKβ^SSEE^-mediated induction of osteoclastogenesis requires NF-κB-mediated gene regulation.

### Constitutively active IKKβ is sufficient for the establishment of in vivo osteolysis

**Fig. 6:**
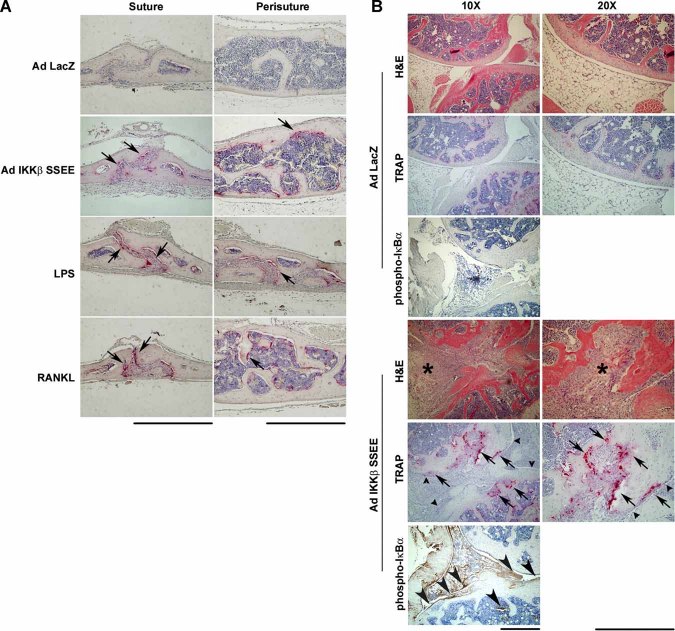
Active IKKβ is sufficient for osteolysis. (*A*) Images of TRACP-stained histologic slides of calvarial bones of mice injected supracalvarially with adenovirus expressing LacZ (Ad LacZ) or IKKβ^SSEE^ (Ad IKKβ^SSEE^), LPS, or RANKL. Arrows indicate areas of osteoclastic bone erosion. Scale bars = 200 µm. (*B*) Images of H&E-, TRACP-, and immunoperoxidase-phospho-IκBα−stained histologic slides of knees of mice injected intraarticularly with adenovirus expressing LacZ (Ad LacZ) or IKKβ^SSEE^ (Ad IKKβ^SSEE^). Arrows indicate areas of pathologic osteoclastic bone erosion (*pink*) at the articular surface. Asterisks denote areas of potential inflammatory cell infiltrate into the synovial space. Arrowheads in TRACP-stained sections point to remnants of articular cartilage. Arrowheads in lower panel show positive stain for phosphorylated IκBα. Objective used for capturing image is labeled above panels. Scale bars = 200 µm.

To determine the relevance of IKKβ hyperactivation in vivo, we injected mice with adenovirus expressing IKKβ^SSEE^ or LacZ supracalvarially or intraarticularly into the knee joint (Supplemental Fig. 1). While LacZ did not induce an osteoclast response in either calvariae or knees, IKKβ^SSEE^ stimulated a massive local osteolytic response in both settings characterized by bone destruction and the appearance of osteoclasts at sites of bone erosion (Fig. 6*A, B*). To support the role of the kinase activity of IKKβ in mediating this effect, joints injected with adenoviral IKKβ^SSEE^ showed intense immunostaining for phosphorylated IκBα at sites of osteoclastic articular bone erosion, whereas LacZ-infected knees stained negatively for articular osteoclasts and phosphorylated IκBα (Fig. 6*B*).

## Discussion

We provide evidence that osteoclast differentiation can be triggered by an autonomous intracellular signal downstream yet independent of RANK. IKK has been implicated in RANKL-induced osteoclastogenesis,(17–21) but the sufficiency of this single enzyme to independently induce osteoclastogenesis is surprising. The explanation for this phenomenon is likely to involve complex signal regulation that mimics NF-κB activation by RANKL. It is also possible that IKKβ^SSEE^ takes on functions not performed by IKKβ in normal settings. In support of this, we observe that infection of monocytes with IKKβ^SSEE^ results in activation of p100 NF-κB (JO and YA, unpublished observations), which is usually considered to be a function of IKKα.(38) Perhaps atypical functions such as this contribute to its osteoclastogenic activity. Nevertheless, the ability of IKKβ^SSEE^ to induce the osteoclast depends on kinase activity because mutation of the ATP-binding lysine to methionine in the kinase domain abrogated the IKKβ^SSEE^ osteoclastogenic function (Fig. 1*E* and Supplemental Fig. 2).

Differentiation of the osteoclast requires NF-κB.(11) To determine whether the phenotype we observed also requires NF-κB, we tested the ability of IKKβ^SSEE^ to drive osteoclastogenesis in *NF-κB1/2* double-knockout monocytes, in which it failed. In addition to NF-κB, other transcription factors may play a role in the IKKβ^SSEE^ effect. Interestingly, we observed expression of NFATc1 protein, the master regulator of osteoclastogenesis,(39) induced by IKKβ^SSEE^ in monocytes (Supplemental Fig. 3). Whether NFATc1 is required for osteoclastogenesis induced by active IKKβ or whether IKKβ controls NFATc1 activity directly in the differentiating osteoclast is unknown.

NF-κB is also a critical regulator of inflammatory signals,(40) and inflammatory cytokines enhance osteoclast function.(41,42) Since tumor necrosis factor α (TNF-α) and interleukin 1 (IL-1) induce osteoclast differentiation in certain settings,(43) and since we observed both TNF-α and IL-1β expression by monocytes transduced with IKKβ^SSEE^ (JO and YA, unpublished observations), we sought to determine whether these inflammatory factors were required for IKKβ^SSEE^ to induce osteoclast differentiation. Using IKKβ^SSEE^-transduced *TNF-α* or *IL-1 receptor* knockout monocytes, we found that TNF-α and IL-1 are not required for IKKβ^SSEE^ to accomplish its effect in osteoclast differentiation (JO and YA, unpublished observations). Therefore, IKKβ^SSEE^- induced osteoclastogenesis in vitro is uncoupled from inflammatory signaling with respect to TNF-α and IL-1. Nevertheless, given that IKKβ^SSEE^ does induce secretion of these factors, we must consider the possibility that this kinase modulates osteoclast activation at sites of inflammation through inflammatory signals, the nature of which will be investigated in future studies.

Consistent with our in vitro findings, adenoviral gene-transfer experiments revealed that IKKβ^SSEE^ is sufficient for the establishment of osteolysis in vivo. The clinical significance of our findings is highlighted by our observations that IKKβ^SSEE^-induced osteoclastogenesis is refractory to intervention with OPG and deletion of RANK/RANKL. In this regard, a number of conditions in human patients are associated with heightened bone turnover in the setting of inflammation for which a cause has not been identified.(44) Given the potency with which activated IKKβ induces osteoclast appearance and bone destruction in this model, it is important to consider IKKβ activation as an independent cause and a target in therapy for conditions of inflammatory bone destruction.

Our data highlight the critical role of IKKβ in osteoclast differentiation and osteolysis. We have found that constitutively active IKKβ unfolds the osteoclast program in the absence of upstream signals. We report the first evidence of RANK-independent osteoclast differentiation that is induced through a single kinase, and we propose that hyperactivation of human IKKβ may lead to diseases resulting in bone destruction that would be refractory to treatments targeting receptor–proximal signaling molecules.
