# Supplementary figures and images for: IKKβ Activation Is Sufficient for RANK-Independent Osteoclast Differentiation and Osteolysis
Source: J Bone Miner Res. 2010 Feb 1;25(6):1282–94. doi: 10.1002/jbmr.4 (PMC3153134; doi:10.1002/jbmr.4)

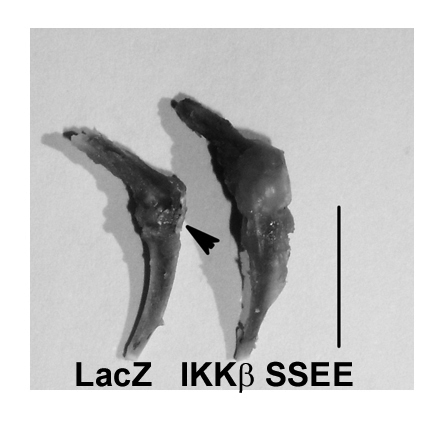

Supplement: Supplementary file 1 [file jbmr0025-1282-SD1.tif]

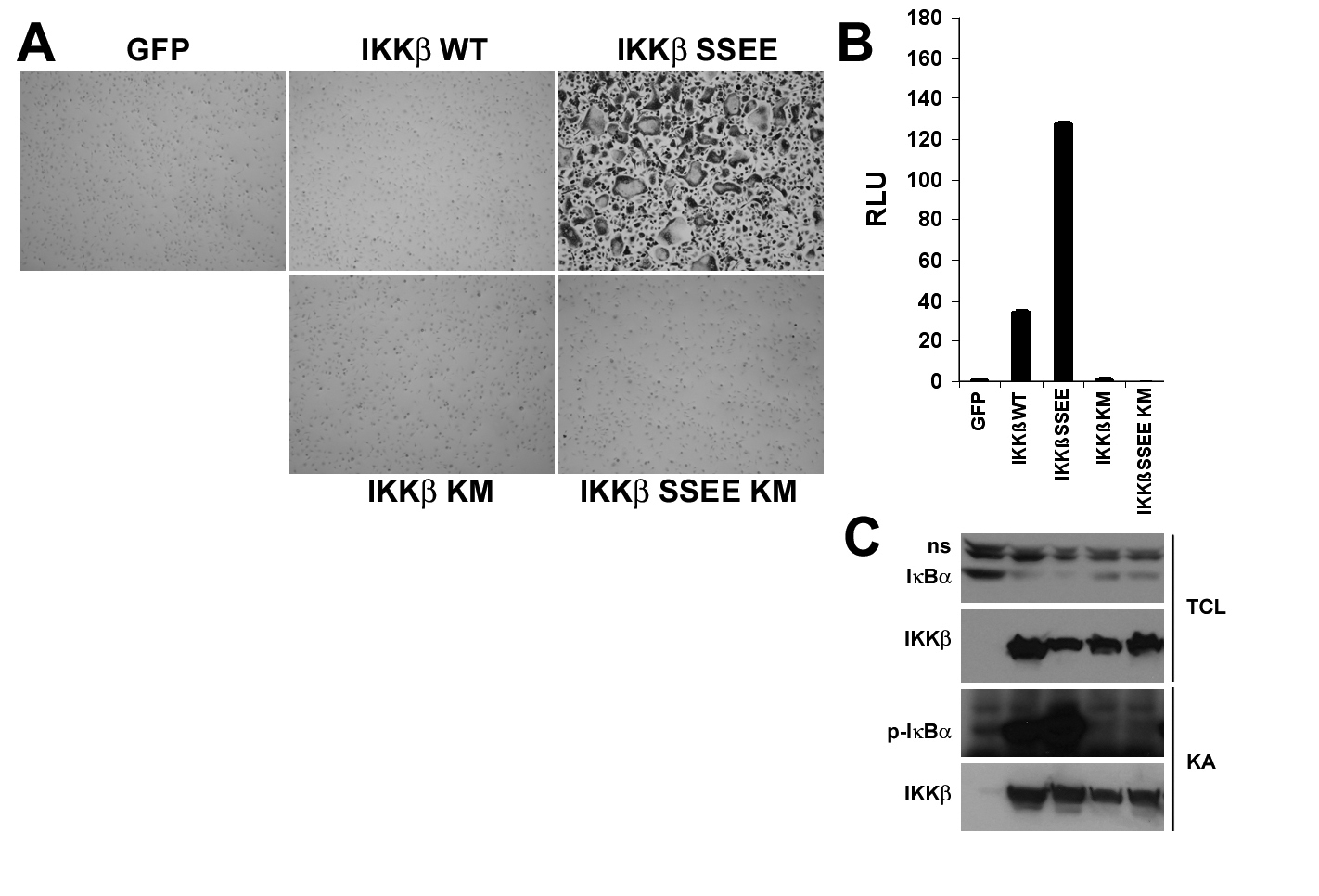

Supplement: Supplementary file 2 [file jbmr0025-1282-SD2.tif]

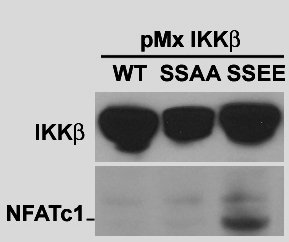

Supplement: Supplementary file 3 [file jbmr0025-1282-SD3.tif]
